# Preoperative Changes in Hematological Markers and Predictors of Glioma Grade and Survival

**DOI:** 10.3389/fphar.2018.00886

**Published:** 2018-08-14

**Authors:** Peng-Fei Wang, Zhe Meng, Hong-Wang Song, Kun Yao, Ze-Jun Duan, Chun-Jiang Yu, Shou-Wei Li, Chang-Xiang Yan

**Affiliations:** ^1^Department of Neurosurgery, Sanbo Brain Hospital, Capital Medical University, Beijing, China; ^2^Department of Pathology, Sanbo Brain Hospital, Capital Medical University, Beijing, China

**Keywords:** glioma, prognostic biomarkers, grades, gender, IDH mutations

## Abstract

**Background:** Preoperative hematological markers that indicate nutritional, coagulation, and inflammation statuses have prognostic value for gliomas. This study aimed to investigate hematological markers with regard to tumor grades, isocitrate dehydrogenase mutations (IDH), age, and sex in patients with gliomas.

**Methods:** From 2008 to 2017, patients with a pathological diagnosis of glioma who underwent surgery were retrospectively enrolled in this study. Information from clinical records, including age, sex, preoperative experiment tests (routine blood tests, biochemistry, and coagulation examinations), pathological results, and IDH status, was collected. A univariable survival analysis was performed. Hematological factors such as the neutrophil-to-lymphocyte ratio (NLR), platelet-to-lymphocyte-ratio (PLR), and albumin-to-globulin (AGR) were calculated. The prognostic nutrition index (PNI) was calculated as 10 × serum albumin value (g/dl) + 0.005 × peripheral lymphocyte count (per mm^3^).

**Results:** Our study included 706 patients. The univariate analysis showed that age, IDH-1, and hematological factors were all significantly associated with overall survival (OS) in patients with gliomas. Our results showed that inflammation markers (NLR, PLR, and fibrinogen) were positively associated with age, whereas AGR was negatively associated with age. The PLR was significantly increased, whereas the AGR and PNI were decreased in women with gliomas, as compared with men. We found that inflammation markers increased and nutrition markers decreased with gliomas grade. However, these hematological markers did not significantly differ with IDH status. NLR was the best single hematological marker for distinguishing glioblastoma (GBM) [0.684 (0.645–0.723)], IDH-wt GBM [0.672 (0.631–0.71)] from other gliomas subtypes. Combinations of age with PNI and age with AGR were the best predictors of GBM [0.750 (0.713–0.786)] and IDH-wt GBM [0.759 (0.719–0.798)], respectively.

**Conclusion:** Preoperative hematological marker levels vary among glioma grades and have high predictive values for GBM.

## Introduction

Gliomas are the most common malignant primary tumors that originate from the central nervous systems (CNS) (Ostrom et al., 2017). According to the 2016 World Health Organization (WHO) criteria, gliomas are classified as grades I-IV, with different molecular subtypes and histopathologies (Louis et al., 2016). Glioma therapies greatly vary across the different grades and molecular subtypes (Tanase et al., 2015; Nabors et al., 2017). Designing individual therapies after identifying the glioma grade and molecular subtype is very helpful. However, the traditional way to identify the glioma grade and molecular subtype is to acquire tumor tissue by surgery or biopsy, which causes significant trauma to the human body. The disadvantages of conventional invasive methods motivate us to develop a reliable method for predicting glioma grade and survival.

Recently, mounting evidence has suggested that preoperative hematological markers related to nutrition, coagulation, and inflammation are predictive and prognostic factors of cancers (Perisanidis et al., 2015; He J. et al., 2017; Hwang et al., 2017; Ye et al., 2018; Zhao et al., 2018). In gliomas, neutrophil-to-lymphocyte ratio (NLR) (Han et al., 2015; Lopes et al., 2018), platelet-to-lymphocyte ratio (PLR) (Han et al., 2015), fibrinogen, albumin-to-globulin ratio (AGR) (Xu W. Z. et al., 2017), and prognostic nutrition index (PNI) (He Z. Q. et al., 2017; Xu W. Z. et al., 2017) have been identified as prognostic markers. Moreover, the levels of these hematological markers vary among tumor grades, providing diagnostic value for gliomas (Schwartzbaum et al., 1999; Zadora et al., 2015; Zheng et al., 2017). However, existing studies that investigated their diagnostic values have not included all markers. Little is known about the relationship between age, sex, and the hematological factors for the prognoses of patients with gliomas.

The present study aimed to identify the tumor grade, subtype, and clinical outcome in gliomas using hematological markers. We compared the levels of these hematological markers, including NLR, PLR, fibrinogen, AGR, and PNI among patients with different glioma grades and molecular subtypes. We also performed a receiver operating characteristic curve (ROC) analysis to identify the optimal combinations for glioma diagnosis.

## Methods

### Study population

We retrospectively enrolled 706 patients with gliomas at the Sanbo Brain Hospital, from 2008 to 2017. All patients underwent surgical treatment and had a pathological diagnosis of glioma (Louis et al., 2007, 2016). Routine biochemistry and coagulation blood test results were obtained preoperatively. Patients with metabolic diseases, hematological diseases, autoimmune diseases, or current infections were excluded. Patients who were treated with glucocorticoids or anti-inflammatory drugs were also not included. IDH-1R^132H^ mutations were determined by immunohistochemistry, as described in our previous report (Wang et al., 2017). Overall survival (OS) was calculated from the date of surgery to death or censored at the final follow-up, which was in December 2017. This study was approved by the ethics committee of the Sanbo Brain Hospital, Capital Medical University.

### Calculations of hematological markers

We calculated the hematological markers as follows: NLR = neutrophil count/lymphocyte count; PLR = platelet count/lymphocyte count; AGR = albumin/globulin; and PNI = albumin [g/L] + total lymphocyte count × 5.

### Statistical analysis

SPSS 22.0, Graph Pad Prism 6, and X-tile 3.61 were used for data analysis, drawing figures, and identifying continuous variable cutoffs, respectively. The hematological marker levels were compared across tumor grades, IDH-1 subtypes, and sexes using the student's *t-*test. The correlation of hematological markers with age was analyzed with the *spearman test*. The *area under the curve (AUC)* was acquired using ROC analysis, to identify the diagnostic value of prognostic factors and their combinations. A *univariate analysis* was used for the survival analysis. Statistical significance was determined as *p* < 0.05.

## Results

### Study characteristics

The basic clinical characteristics of the 706 patients with gliomas are shown in Table 1. The median patient age was 45 (7–80) years, and 42.4% (299/706) of the patients were women. There were 238 (33.7%), 154 (21.8%), and 314 (44.5%) patients with grade II, III, and IV gliomas, respectively. The IDH-1 mutation rate was 44.1% (311/706). The mean neutrophil, platelet, lymphocyte, albumin, and fibrinogen values were 3.83 ± 1.38, 222.64 ± 58.90, 1.87 ± 0.62, 43.03 ± 3.96, and 2.44 ± 0.61, respectively. The mean NLR, PLR, AGR, and PNI were 2.39 ± 1.71, 133.26 ± 62.44, 1.87 ± 0.36, and 52.37 ± 5.27, respectively. The survival analysis showed that younger age; IDH-1 mutations; higher albumin, AGR, and PNI levels; and lower NLR, PLR, and fibrinogen levels were favorable prognostic factors of OS in all gliomas and glioblastomas (GBMs) (Table 2).

**Table 1:** Baseline characteristics.

**Age**	
Mean ± SD	45.15 ± 13.39
Median (range)	45 (7-80)
**Gender**	
Female	299 (42.4%)
Male	407 (57.6%)
**Tumor grade**	
WHO II	238 (33.7%)
WHO III	154 (21.8%)
WHO IV	314 (44.5%)
**IDH-1^R132H^**	
Mutation	311 (44.1%)
Wild type	395 (55.9%)
**Neutrophil (10^9^/L)**	
Mean ± SD	3.83 ± 1.38
**Platelet (10^9^/L)**	
Mean ± SD	222.64 ± 58.90
**Lymphocyte (10^9^/L)**	
Mean ± SD	1.87 ± 0.62
**NLR**	
Mean ± SD	2.39 ± 1.71
**PLR**	
Mean ± SD	133.26 ± 62.44
**Fibrinogen (g/l)**	
Mean ± SD	2.44 ± 0.61
**Albumin (g/L)**	
Mean ± SD	43.03 ± 3.96
**AGR**	
Mean ± SD	1.87 ± 0.36
**PNI**	
Mean ± SD	52.37 ± 5.27

*Data are n (%), unless otherwise indicated. SD, standard deviation; IDH, isocitrate dehydrogenase; NLR, neutrophil-to-lymphocyte ratio; PLR, platelet-to-lymphocyte ratio; AGR, albumin-to-globulin ratio; PNI, prognostic nutrition index*.

**Table 2:** Univariate analysis of prognostic factors.

**Variable**	**All gliomas**		**GBM**	
	**HR (95% CI)**	***P***	**HR (95% CI)**	***P***
Age	1.04 (1.03–1.05)	0.000	1.01 (1.00–1.03)	0.019
Gender	1.04 (0.82–1.31)	0.770	0.96 (0.72–1.28)	0.782
IDH-1^R132H^	3.25 (2.50–4.23)	0.000	1.75 (1.22–2.50)	0.002
NLR	0.41 (0.31–0.54)	0.000	0.57 (0.41–0.79)	0.001
PLR	0.51 (0.36–0.71)	0.000	0.69 (0.50–0.93)	0.017
Fib	0.37 (0.26–0.52)	0.000	0.59 (0.41–0.83)	0.003
Alb	1.62 (1.24–2.13)	0.000	0.46 (0.25–0.84)	0.012
AGR	2.00 (1.47–2.73)	0.000	1.51 (1.03–2.21)	0.036
PNI	1.69 (1.33–2.14)	0.000	1.73 (1.08–2.76)	0.022

### The association of hematological markers with age and sex in all gliomas and GBM

We found that nutrition-related markers, such as albumin, AGR, and PNI, were significantly negatively correlated with age in all gliomas (*p* < 0.001) and GBMs (*p* < 0.001). In contrast, a positive correlation was observed with age and NLR (*p* < 0.001), PLR (*p* = 0.023), and fibrinogens (*p* < 0.001) in all gliomas. Although fibrinogen (*p* < 0.001) positively correlated with age, a non-statistically significant association was found between age and NLR (*p* = 0.050) and PLR (*p* = 0.256) in GBMs. These results indicate generally poor nutrition and severe inflammation states in elderly patients with gliomas (Table 3).

**Table 3:** The association of hematological factors with age and gender in gliomas.

**Variable**	**All glioma**					**GBM**				
	**Age**		**Gender**			**Age**		**Gender**	
	**Correlation (*r*)**	***P***	**Female**	**Male**	***P***	**Correlation (*r*)**	***P***	**Female**	**Male**	***P***
NLR	0.142	0.000	2.42 ± 1.88	2.37 ± 1.57	0.745	0.111	0.050	3.01 ± 2.35	2.71 ± 1.39	0.206
PLR	0.086	0.023	143.56 ± 71.23	125.70 ± 53.95	0.000	0.064	0.256	158.25 ± 81.51	133.06 ± 55.77	0.003
Fib	0.278	0.000	2.47 ± 0.61	2.41 ± 0.61	0.244	0.232	0.000	2.68 ± 0.68	2.57 ± 0.69	0.161
Alb	−0.185	0.000	42.65 ± 3.86	43.31 ± 4.02	0.029	−0.198	0.000	41.87 ± 4.13	42.39 ± 4.09	0.275
AGR	−0.264	0.000	1.79 ± 0.35	1.92 ± 0.35	0.000	−0.253	0.000	1.67 ± 0.35	1.82 ± 0.37	0.000
PNI	−0.229	0.000	51.76 ± 5.32	52.81 ± 5.19	0.009	−0.224	0.000	50.17 ± 5.51	51.19 ± 4.85	0.084

Increased levels of albumin (43.31 ± 4.02 vs. 42.65 ± 3.86, *p* = 0.029), AGR (1.92 ± 0.35 vs. 1.79 ± 0.35, *p* = 0.000), and PNI (52.81 ± 5.19 vs. 51.76 ± 5.32, *p* = 0.009) were found in men, in contrast to women with gliomas. However, only one nutrition marker, AGR, was higher in men than in women with GBMs (1.82 ± 0.37 vs. 1.67 ± 0.35, *p* = 0.000). PLRs significantly decreased in men in both all glioma (143.56 ± 71.23 vs. 125.70 ± 53.95, *p* = 0.000) and GBM (158.25 ± 81.51 vs. 133.06 ± 55.77, *p* = 0.003; Table 3) cohorts.

### Hematological marker levels vary among glioma grades and molecular subtypes

**Figure 1:**
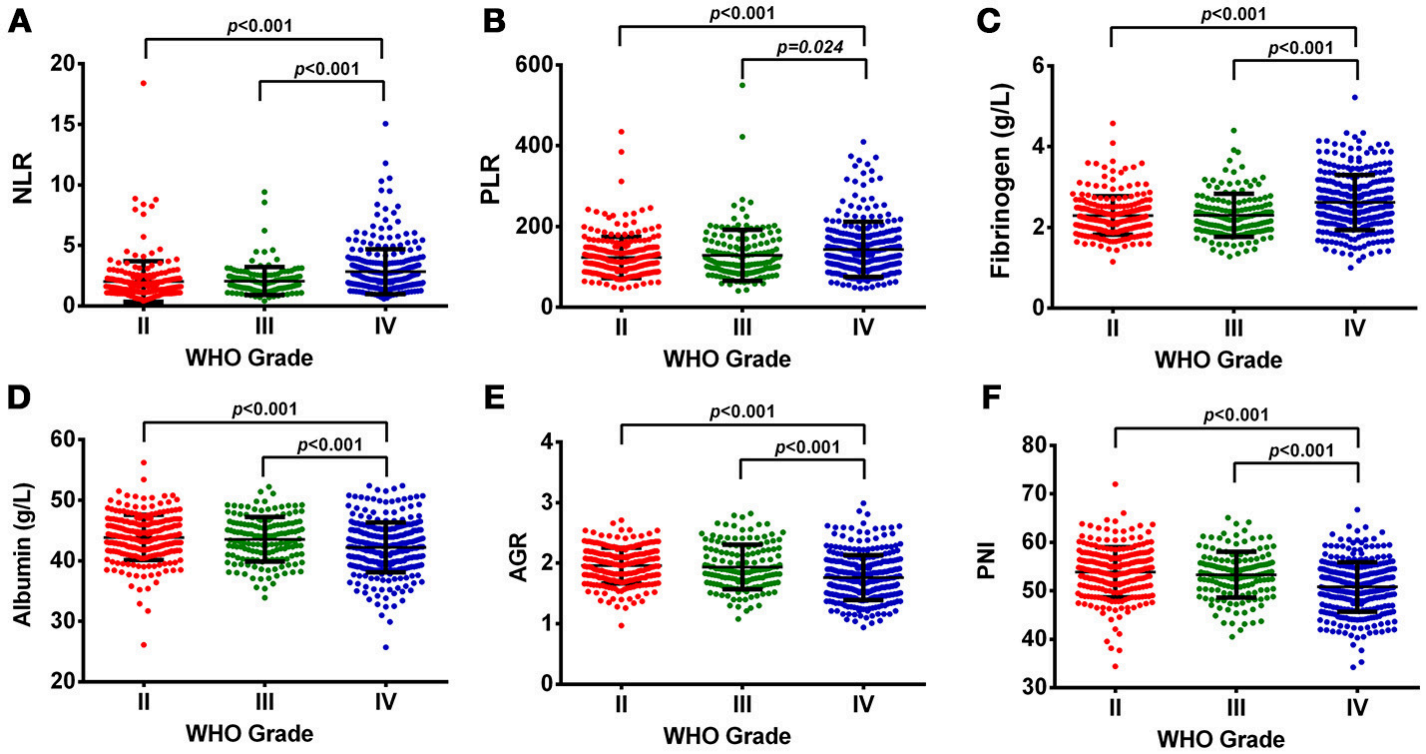
**(A)** The diversity of NLR in different glioma grades. **(B)** The diversity of PLR in different glioma grades. **(C)** The diversity of fibrinogen in different glioma grades. **(D)** The diversity of albumin in different glioma grades. **(E)** The diversity of AGR in different glioma grades. **(F)** The diversity of PNI in different glioma grades.

**Figure 2:**
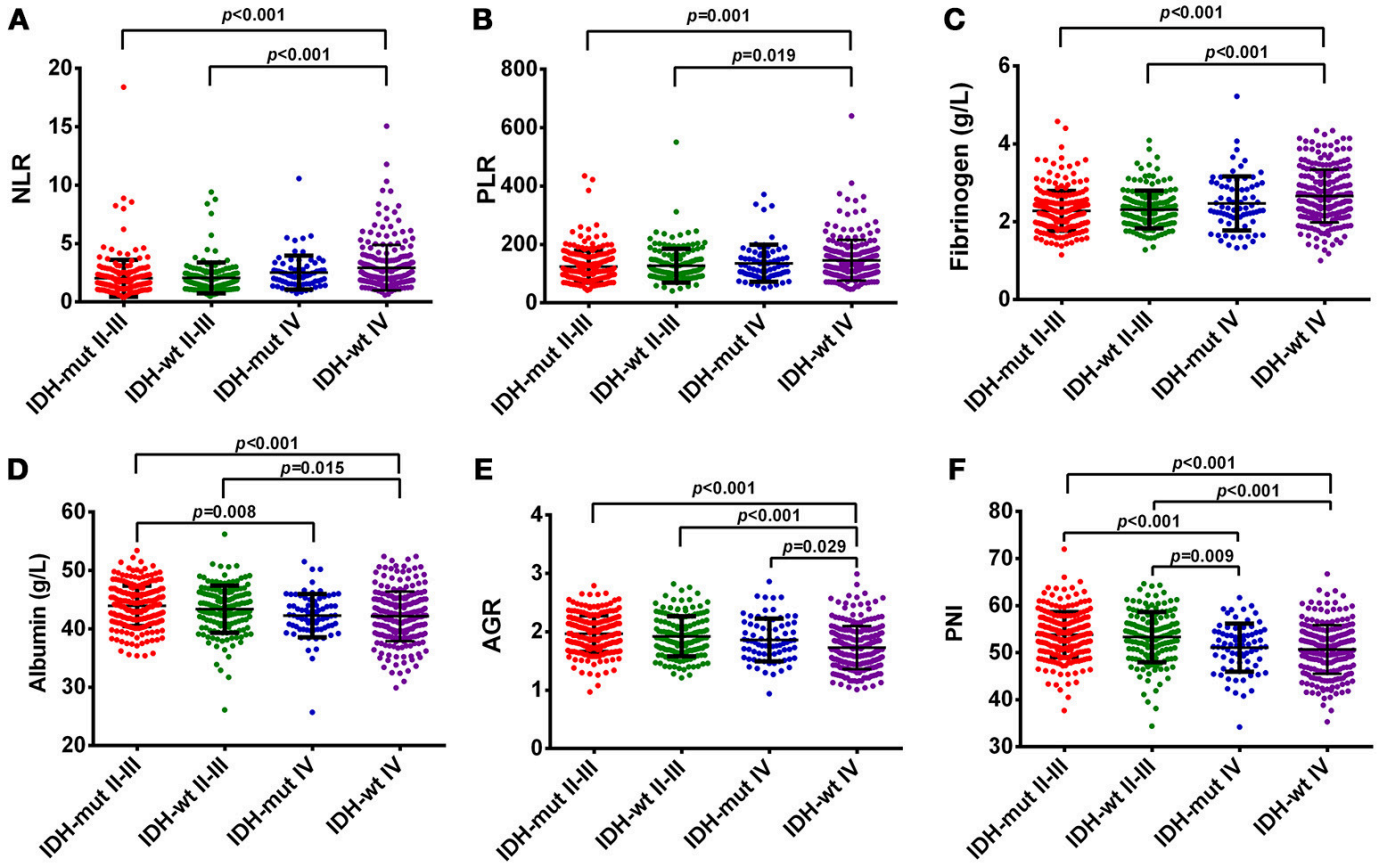
**(A)** The diversity of NLR in different glioma grades and IDH-mutation status. **(B)** The diversity of PLR in different glioma grades and IDH-mutation status. **(C)** The diversity of fibrinogen in different glioma grades and IDH-mutation status. **(D)** The diversity of albumin in different glioma grades and IDH-mutation status. **(E)** The diversity of AGR in different glioma grades and IDH-mutation status. **(F)** The diversity of PNI in different glioma grades and IDH-mutation status.

In our study, OS was negatively correlated with glioma grades (*p* < 0.001, Supplementary Figure 1A). Increased levels of NLR, PLR, and fibrinogen were noted in GBMs, compared with Grade II and III gliomas (Figure 1). The levels of albumin, AGR, and PNI significantly decreased in GBMs, in contrast to Grade III and II gliomas (Figure 1). Next, we classified gliomas based on IDH-1^R132H^ status as follows: IDH-mut II-III, IDH-wt II-III, IDH-mut GBM, and IDH-wt GBM. OS significantly differed among the four groups (*p* < 0.001, Supplementary Figure 1B). Inflammatory markers such as NLR, PLR, and fibrinogen were elevated in IDH-wt GBM, compared with II-III gliomas with or without IDH mutation. However, the levels of nutrition markers varied among the four subtypes (Figure 2). The worst nutritional status was observed in IDH-wt GBM, followed by that of IDH-mut GBM (Figure 2).

### The diagnostic value of hematological markers in predicting glioma grade and subtype

**Figure 3:**
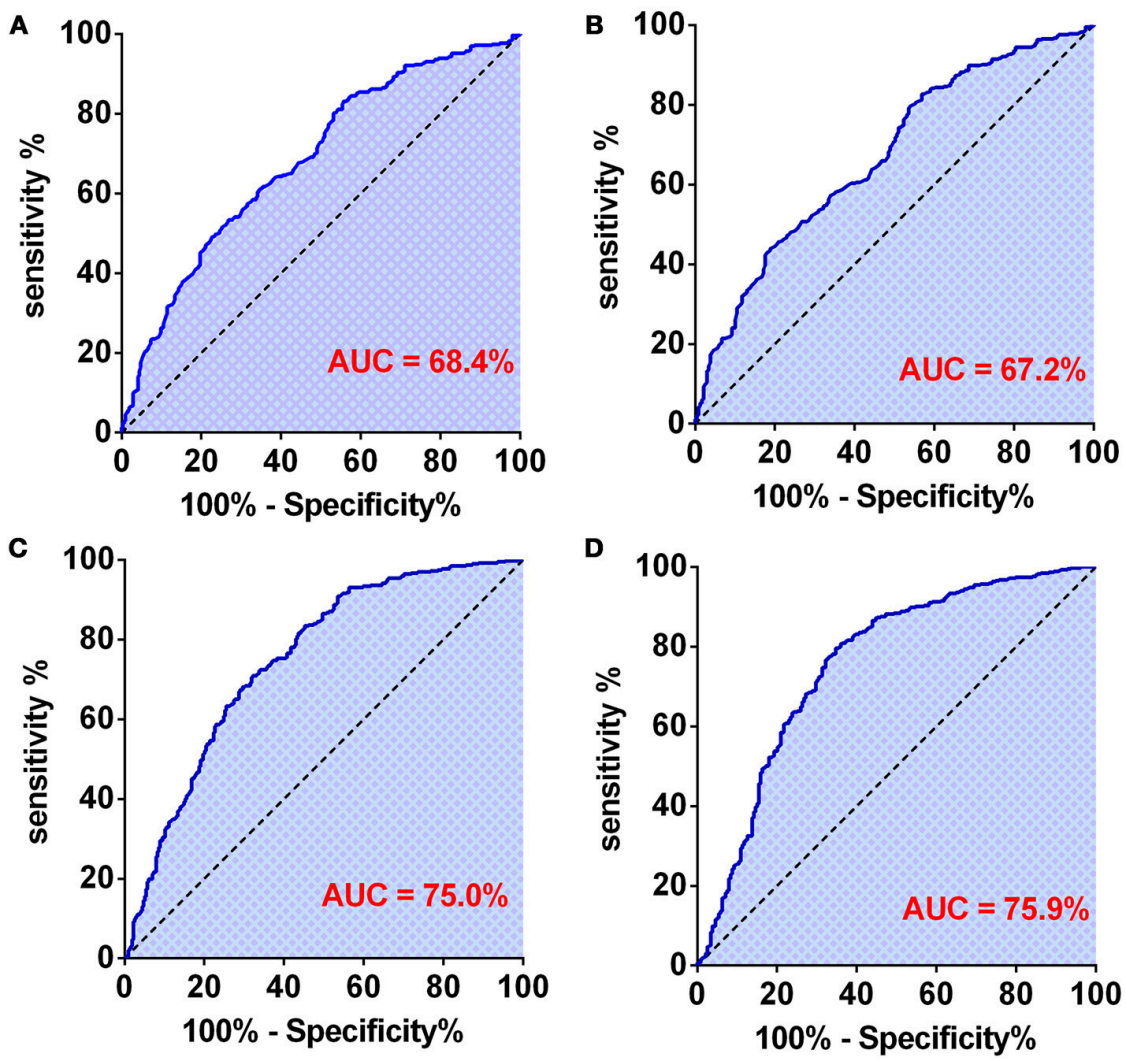
**(A)** The diagnostic value of preoperative NLR in patients with GBM. **(B)** The diagnostic value of preoperative NLR in patients with grade IV-IDH wild type. **(C)** The diagnostic value of preoperative age + PNI combination in patients with GBM. **(D)** The diagnostic value of preoperative age + AGR combination in patients with grade IV-IDH wild type.

First, we used one marker to predict GBMs and IDH-1 wt subtype (Table 4). The ROC curve analysis showed that NLR had the highest diagnostic value for distinguishing GBM from grade II to III [0.684 (0.645–0.723), Figure 3A] and predicting the GBM IDH-1 wt molecular subtype [0.672 (0.631–0.71), Figure 3B]. Next, we investigated whether we could increase the diagnostic value by combining these hematological markers (Table 4). We found that the combination of age and PNI was best suited for predicting the diagnosis of GBMs [0.750 (0.713–0.786)] (Figure 3C). Furthermore, the combination of age and AGR had the highest AUC for distinguishing GBM IDH-1 wt from other subtypes [0.759 (0.719–0.798), Figure 3D].

**Table 4:** The diagnostic value of hematological markers in predicting glioma grade and subtype.

**Marker**	**AUC (95% CI)**	
	**GBM**	**IDH – wt GBM**
NLR	0.684 (0.645–0.723)	0.672 (0.631–0.714)
PLR	0.590 (0.547–0.632)	0.594 (0.550–0.638)
Fib	0.652 (0.611–0.693)	0.660 (0.616–0.703)
Alb	0.618 (0.576–0.660)	0.602 (0.557–0.647)
AGR	0.659 (0.619–0.700)	0.670 (0.627–0.713)
PNI	0.661 (0.621–0.701)	0.645 (0.602–0.688)
Age + NLR	0.745(0.708–0.782)	0.753(0.713–0.793)
Age + PLR	0.727(0.689–0.765)	0.742(0.701–0.783)
Age + Fib	0.733(0.695–0.771)	0.746(0.705–0.786)
Age + Alb	0.733(0.695–0.770)	0.742(0.702–0.783)
Age + AGR	0.738(0.701–0.776)	0.759(0.719–0.798)
Age + PNI	0.750(0.713–0.786)	0.753(0.713–0.793)
NLR + PLR	0.680 (0.641–0.720)	0.666 (0.623–0.708)
NLR + Fib	0.689 (0.649–0.729)	0.688 (0.645–0.730)
NLR + Alb	0.699 (0.661–0.738)	0.688 (0.647–0.730)
NLR+ AGR	0.712 (0.672–0.751)	0.711 (0.669–0.753)
NLR + PNI	0.692 (0.653–0.731)	0.683 (0.641–0.724)
PLR + Fib	0.665 (0.624–0.706)	0.673 (0.630–0.717)
PLR + Alb	0.647 (0.606–0.688)	0.639 (0.595–0.682)
PLR + AGR	0.676 (0.635–0.716)	0.685 (0.643–0.727)
PLR + PNI	0.662 (0.622–0.702)	0.650 (0.607–0.692)
Fib + Alb	0.678 (0.638–0.718)	0.670 (0.627–0.713)
Fib + AGR	0.681 (0.641–0.721)	0.694 (0.652–0.736)
Fib + PNI	0.704 (0.665–0.743)	0.693 (0.651–0.735)
Alb + AGR	0.664 (0.623–0.704)	0.671 (0.628–0.714)
Alb + PNI	0.660 (0.620–0.700)	0.646 (0.604–0.689)
AGR + PNI	0.690 (0.650–0.729)	0.689 (0.647–0.731)

## Discussion

Our study shows that the prognostic value of hematological markers and their levels vary among glioma grades and molecular subtypes. Moreover, patient age, sex, and hematological markers were strongly associated. These results indicate different inflammation and nutritional states, with regard to patient age, sex, tumor grade, and genetic alternations. Furthermore, we combined these prognostic factors to predict glioma classification.

This study shows that NLR, PLR, and fibrinogens were positively correlated with glioma grade, whereas PNI and AGR were negatively correlated with glioma grade. In addition, previous studies observed higher NLRs (Zadora et al., 2015) and lower PNIs in higher grade gliomas (He Z. Q. et al., 2017). These results indicate that patients with higher grade gliomas have more severe inflammation and poorer nutritional statuses. NLR and PLR hematological markers were not associated with IDH mutations in this study, which is consistent with our previous report (Wang et al., 2017). However, immune responses in the tumor microenvironment are more significantly regulated by IDH mutations in lower grade gliomas (Qian et al., 2018). This result might be due to differences between systemic and local inflammation and immune regulation.

We found that inflammation markers were positively correlated with age in patients with gliomas or GBM. These results explain why older age is a negative prognostic factor in gliomas, as a result of inflammation status. Furthermore, we observed different hematological marker levels between women and men, and specifically, albumin was higher in men than in women. Consistent with data from other studies (Zhou et al., 2016), we observed that PNI was higher in men than women. We found that PLR was higher in women than in men, both in gliomas and GBMs, which was contrary to Xu et al.'s report (Xu W. et al., 2017). There were no survival differences with regard to sex, and this finding might be influenced by NLR and fibrinogens, which showed no differences with respect to sex.

We observed that NLR was the best single predictive marker for distinguishing GBM and IDH-wt GBM from other types of gliomas. Our results are consistent with the finding that NLR was able to predict the diagnosis of glioma from acoustic neuroma, meningioma, and non-lesional epilepsy (Zheng et al., 2017). Our study showed that the best combinations for predicting the diagnosis of GBM or IDH-wt GBM were age + PNI and age + AGR; however, NLR + LMR had the highest diagnostic accuracy. It should be noted that grade I gliomas were included in a previous study (Zheng et al., 2017), and more studies are needed to confirm and optimize the prognostic factor combinations.

The mechanisms underlying the associations between hematological markers and glioma grades were not very clear. The tumor-infiltrating neutrophil count was positively correlated with glioma grade, by promoting the progression of glioma (Fossati et al., 1999; Liang et al., 2014). Moreover, Fossati et al. observed that circulating neutrophils were positively associated with glioma grade, which was influenced by glioma-derived factors that may impact neutrophil circulation and infiltration (Fossati et al., 1999). Neutrophil-induced immunosuppression and angiogenesis in gliomas have also been found to promote glioma progression (Massara et al., 2017). The circulating fibrinogen could activate neutrophils via integrin α_M_β_2_ (Steinbrecher et al., 2010; Massara et al., 2017). Natural killer cell function has also been found to be negatively affected by fibrinogen, which suppresses anti-tumor immunity (Degen and Palumbo, 2012). Albumin, AGR, and PNI are all associated with nutritional status in patients with cancer. Albumin also reflects the systemic inflammation status, as it is downregulated by tumor necrosis factor alpha (TNFα) and interleukin 6 (IL-6) (Chojkier, 2005). Furthermore, TNFα and IL-6 could negatively affect the function of immune cells in GBMs (Kozlowska et al., 2016).

This study has several limitations. First, as our study had a retrospective design, some unavoidable biases may exist. Second, we did not continuously monitor various prognostic factors. Furthermore, we only investigated the changes of blood indices and did not study the differences of tumor immune microenvironments. Therefore, prospective multicenter studies, continuous perioperative monitoring, and further molecular biology experiments are needed. Furthermore, previous reports indicated that hematological markers could effectively distinguish gliomas from non-lesional epilepsy, acoustic neuroma, and meningioma (Zheng et al., 2017). However, the present study focused on the correlation between hematological markers and tumor grade, molecular subtype, and clinical outcomes in glioma. Therefore, we did not investigate the hematological markers in other neuropathological states.

In conclusion, our study proves that there are different prognostic factors among glioma grades and molecular subtypes, and NLR was the best single marker to distinguish GBM and IDH-wt GBM. The combinations of age with PNI and age with AGR could best diagnose IDH-wt GBM. These prognostic factors correlate with age and sex in patients with gliomas.

## Author contributions

C-XY and S-WL: conception and design; P-FW, ZM, and H-WS: collection and follow-up; P-FW and ZM: data analysis and interpretation. All authors manuscript writing, final approval of manuscript, and accountable for all aspects of the work.

### Conflict of interest statement

The authors declare that the research was conducted in the absence of any commercial or financial relationships that could be construed as a potential conflict of interest.

## Supplementary material

Supplementary Figure 1 **(A)** Kaplan-Meier survival curves of glioma patients in grade II (*n* = 238), III (*n* = 154), IV (*n* = 314), have significant value (*p* < 0.001). **(B)** Kaplan-Meier survival curves of glioma patients in IDH-1^R132H^-mutant II-III (*n* = 236), IDH-1^R132H^-wild type II-III (*n* = 156), IDH-1^R132H^-mutant IV (*n* = 75), IDH-1^R132H^-wild type (*n* = 239) have significant value (*p* < 0.001).

## References

[B1] Chojkier M. (2005). Inhibition of albumin synthesis in chronic diseases: molecular mechanisms. J. Clin. Gastroenterol. 39, S143–S146. 10.1097/01.mcg.0000155514.17715.39 15758650

[B2] Degen J. L. Palumbo J. S. (2012). Hemostatic factors, innate immunity and malignancy. Thromb. Res. 129(Suppl. 1), S1–S5. 10.1016/S0049-3848(12)70143-3 22682116

[B3] Fossati G. Ricevuti G. Edwards S. W. Walker C. Dalton A. Rossi M. L. (1999). Neutrophil infiltration into human gliomas. Acta Neuropathol. 98, 349–354. 10.1007/s004010051093 10502039

[B4] Han S. Liu Y. Li Q. Li Z. Hou H. Wu A. (2015). Pre-treatment neutrophil-to-lymphocyte ratio is associated with neutrophil and T-cell infiltration and predicts clinical outcome in patients with glioblastoma. BMC Cancer 15:617. 10.1186/s12885-015-1629-7 26341881 PMC4559944

[B5] He J. Pan H. Liang W. Xiao D. Chen X. Guo M.. (2017). Prognostic effect of albumin-to-globulin ratio in patients with solid tumors: a systematic review and meta-analysis. J. Cancer 8, 4002–4010. 10.7150/jca.21141 29187875 PMC5706002

[B6] He Z. Q. Ke C. Al-Nahari F. Duan H. Guo C. C. Wang Y.. (2017). Low preoperative prognostic nutritional index predicts poor survival in patients with newly diagnosed high-grade gliomas. J. Neurooncol. 132, 239–247. 10.1007/s11060-016-2361-0 28078639

[B7] Hwang K. T. Chung J. K. Roh E. Y. Kim J. Oh S. Kim Y. A.. (2017). prognostic influence of preoperative fibrinogen to albumin ratio for breast cancer. J. Breast Cancer 20, 254–263. 10.4048/jbc.2017.20.3.254 28970851 PMC5620440

[B8] Kozlowska A. K. Tseng H. C. Kaur K. Topchyan P. Inagaki A. Bui V. T.. (2016). Resistance to cytotoxicity and sustained release of interleukin-6 and interleukin-8 in the presence of decreased interferon-gamma after differentiation of glioblastoma by human natural killer cells. Cancer Immunol. Immunother. 65, 1085–1097. 10.1007/s00262-016-1866-x 27439500 PMC4996719

[B9] Liang J. Piao Y. Holmes L. Fuller G. N. Henry V. Tiao N.. (2014). Neutrophils promote the malignant glioma phenotype through S100A4. Clin. Cancer Res. 20, 187–198. 10.1158/1078-0432.CCR-13-1279 24240114 PMC4422653

[B10] Lopes M. Carvalho B. Vaz R. Linhares P. (2018). Influence of neutrophil-lymphocyte ratio in prognosis of glioblastoma multiforme. J. Neurooncol. 136, 173–180. 10.1007/s11060-017-2641-3 29076002

[B11] Louis D. N. Ohgaki H. Wiestler O. D. Cavenee W. K. Burger P. C. Jouvet A.. (2007). The 2007 WHO classification of tumours of the central nervous system. Acta Neuropathol. 114, 97–109. 10.1007/s00401-007-0243-4 17618441 PMC1929165

[B12] Louis D. N. Perry A. Reifenberger G. von Deimling A. Figarella-Branger D. Cavenee W. K.. (2016). The 2016 World Health Organization classification of tumors of the central nervous system: a summary. Acta Neuropathol. 131, 803–820. 10.1007/s00401-016-1545-1 27157931

[B13] Massara M. Persico P. Bonavita O. Mollica Poeta V. Locati M. Simonelli M.. (2017). Neutrophils in Gliomas. Front. Immunol. 8:1349. 10.3389/fimmu.2017.01349 29123517 PMC5662581

[B14] Nabors L. B. Portnow J. Ammirati M. Baehring J. Brem H. Butowski N.. (2017). NCCN guidelines insights: central nervous system cancers, version 1.2017. J. Natl. Compr. Canc. Netw. 15, 1331–1345. 10.6004/jnccn.2017.0166 29118226

[B15] Ostrom Q. T. Gittleman H. Liao P. Vecchione-Koval T. Wolinsky Y. Kruchko C.. (2017). CBTRUS Statistical report: primary brain and other central nervous system tumors diagnosed in the United States in 2010-2014. Neuro Oncol. 19, v1–v88. 10.1093/neuonc/nox158 29117289 PMC5693142

[B16] Perisanidis C. Psyrri A. Cohen E. E. Engelmann J. Heinze G. Perisanidis B.. (2015). Prognostic role of pretreatment plasma fibrinogen in patients with solid tumors: a systematic review and meta-analysis. Cancer Treat. Rev. 41, 960–970. 10.1016/j.ctrv.2015.10.002 26604093

[B17] Qian Z. Li Y. Fan X. Zhang C. Wang Y. Jiang T.. (2018). Molecular and clinical characterization of IDH associated immune signature in lower-grade gliomas. Oncoimmunology 7:e1434466. 10.1080/2162402X.2018.1434466 29872572 PMC5980422

[B18] Schwartzbaum J. A. Lal P. Evanoff W. Mamrak S. Yates A. Barnett G. H.. (1999). Presurgical serum albumin levels predict survival time from glioblastoma multiforme. J. Neurooncol. 43, 35–41. 10.1023/A:1006269413998 10448869

[B19] Steinbrecher K. A. Horowitz N. A. Blevins E. A. Barney K. A. Shaw M. A. Harmel-Laws E.. (2010). Colitis-associated cancer is dependent on the interplay between the hemostatic and inflammatory systems and supported by integrin alpha(M)beta(2) engagement of fibrinogen. Cancer Res. 70, 2634–2643. 10.1158/0008-5472.CAN-09-3465 20233870 PMC4288842

[B20] Tanase C. Albulescu R. Codrici E. Popescu I. D. Mihai S. Enciu A. M.. (2015). Circulating biomarker panels for targeted therapy in brain tumors. Future Oncol. 11, 511–524. 10.2217/fon.14.238 25241806

[B21] Wang P. F. Song H. W. Cai H. Q. Kong L. W. Yao K. Jiang T.. (2017). Preoperative inflammation markers and IDH mutation status predict glioblastoma patient survival. Oncotarget 8, 50117–50123. 10.18632/oncotarget.15235 28223536 PMC5564834

[B23] Xu W. Wang D. Zheng X. Ou Q. Huang L. (2017). Sex-dependent association of preoperative hematologic markers with glioma grade and progression. J. Neurooncol. 137, 279–287. 10.1007/s11060-017-2714-3 29260361

[B22] Xu W. Z. Li F. Xu Z. K. Chen X. Sun B. Cao J. W.. (2017). Preoperative albumin-to-globulin ratio and prognostic nutrition index predict prognosis for glioblastoma. Onco. Targets. Ther. 10, 725–733. 10.2147/OTT.S127441 28223828 PMC5308575

[B24] Ye L. L. Oei R. W. Kong F. F. Du C. R. Zhai R. P. Ji Q. H.. (2018). The prognostic value of preoperative prognostic nutritional index in patients with hypopharyngeal squamous cell carcinoma: a retrospective study. J. Transl. Med. 16:12. 10.1186/s12967-018-1391-0 29361946 PMC5781337

[B25] Zadora P. Dabrowski W. Czarko K. Smolen A. Kotlinska-Hasiec E. Wiorkowski K.. (2015). Preoperative neutrophil-lymphocyte count ratio helps predict the grade of glial tumor - a pilot study. Neurol. Neurochir. Pol. 49, 41–44. 10.1016/j.pjnns.2014.12.006 25666772

[B26] Zhao Z. Zhao X. Lu J. Xue J. Liu P. Mao H. (2018). Prognostic roles of neutrophil to lymphocyte ratio and platelet to lymphocyte ratio in ovarian cancer: a meta-analysis of retrospective studies. Arch. Gynecol. Obstet. 297, 849–857. 10.1007/s00404-018-4678-8 29368160

[B27] Zheng S. H. Huang J. L. Chen M. Wang B. L. Ou Q. S. Huang S. Y. (2017). Diagnostic value of preoperative inflammatory markers in patients with glioma: a multicenter cohort study. J. Neurosurg. 3, 1–10. 10.3171/2017.3.JNS161648 29099300

[B28] Zhou X. W. Dong H. Yang Y. Luo J. W. Wang X. Liu Y. H.. (2016). Significance of the prognostic nutritional index in patients with glioblastoma: a retrospective study. Clin. Neurol. Neurosurg. 151, 86–91. 10.1016/j.clineuro.2016.10.014 27816892

