# Supplementary figures and images for: Preoperative Changes in Hematological Markers and Predictors of Glioma Grade and Survival
Source: Front Pharmacol. 2018 Aug 14;9:886. doi: 10.3389/fphar.2018.00886 (PMC6103192; doi:10.3389/fphar.2018.00886)

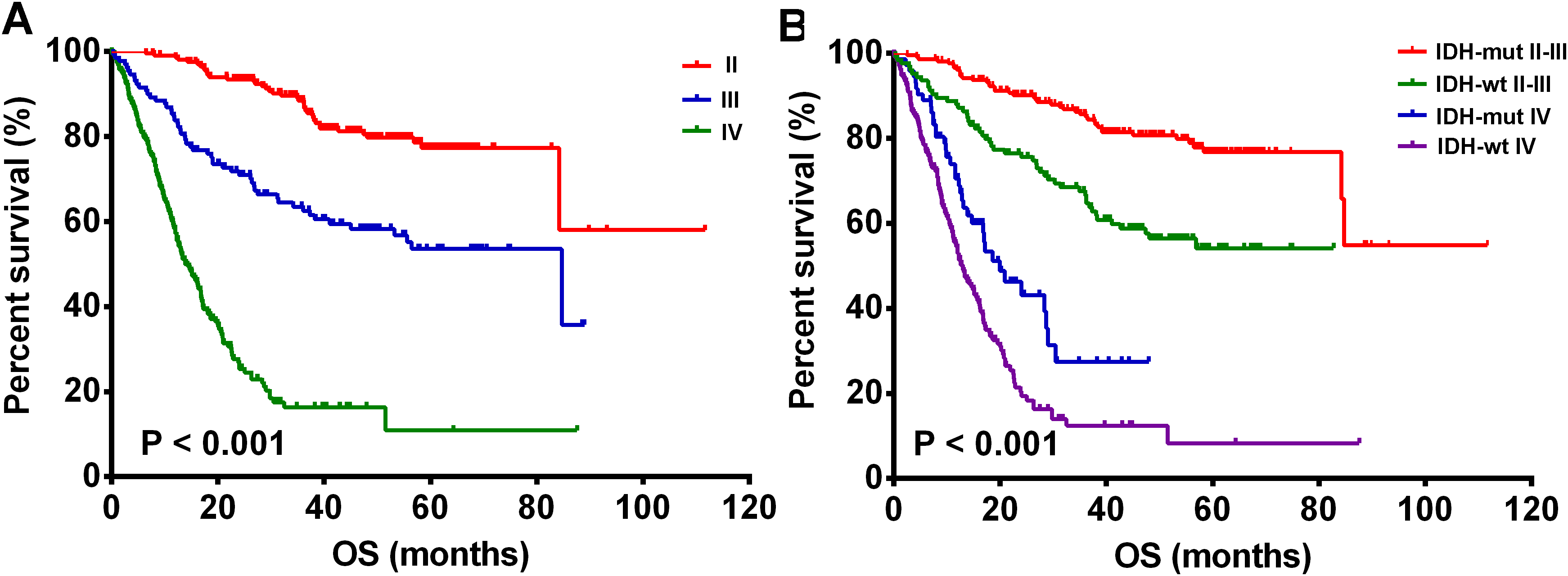

Supplement: Supplementary Figure 1 — (A) Kaplan-Meier survival curves of glioma patients in grade II (n = 238), III (n = 154), IV (n = 314), have significant value (p < 0.001). (B) Kaplan-Meier survival curves of glioma patients in IDH-1R132H-mutant II-III (n = 236), IDH-1R132H-wild type II-III (n = 156), IDH-1R132H-mutant IV (n = 75), IDH-1R132H-wild type (n = 239) have significant value (p < 0.001). [file Image_1.TIF]
